# Memory and potential correlates among children in Jordan

**DOI:** 10.1186/s12888-018-1727-6

**Published:** 2018-05-11

**Authors:** Fidaa Almomani, Nihaya A. Al-sheyab, Murad O. Al-momani, Mazin Alqhazo

**Affiliations:** 1 Department of Rehabilitation Sciences, Faculty of Applied Medical Sciences, Jordan University of Science and Technology, Irbid, 22110 Jordan; 2 Faculty of Nursing, Jordan University of Science and Technology, Irbid, 22110 Jordan; 3 Faculty of Medicine, King Saud University, Riyadh, Saudi Arabia

**Keywords:** Demographics, Interventions, Memory, Leiter international performance scale-revised (Leiter-R), Performance in school

## Abstract

**Background:**

Cognitive functioning hugely affects the performance of tasks of different complexity. Memory, one of the most important cognitive skills, allows children to practice and acquire necessary skills and knowledge and interact with the surrounding environment. Therefore, it is crucial to study the factors that influence the memory of children. The main purpose of his study was to investigate different variables related to memory in school aged children (5–9 years, *N* = 434).

**Methods:**

Parents of the participants provided information about child’s daily behavior, child’s school academic achievement, work and family income data and demographics. Memory skills in children were assessed by using the Leiter International Performance Scale -Revised.

**Results:**

The score of memory increased 2.53 points with upsurge in maternal occupation level, 3.08 points when the child ate breakfast and 4.51 points when the child daily slept nine hours and more. By contrast, increased family income and smoking by father resulted in decreased scores in memory.

**Conclusion:**

Screening for and understanding of memory and relevant factors are vital for broad understanding of children’s capabilities and weaknesses as well as for developing appropriate interventions.

## Background

Different cognitive abilities including reasoning, visualization, memory and attention have huge influence on the performance of tasks of different complexities [1]. Memory is one of the most important cognitive abilities. It is the process of retaining information overtime and the use of past experiences. Memory is essential for learning process and everyday lives. It involves different skills such as encoding, storing, retaining and recalling past information. Individuals would not be able to function in the present or move forward without depending on memory [1, 2].

Memory skills help children to think, plan, reflect, follow direction, imagine and learn basic literacy skills. Memory abilities enhance the process of problem solving in children through the generation of novel ideas and the establishment of cause and effect relationship. In addition, memory helps children to enhance their productivity and creativity, as well as to practice and acquire necessary knowledge and life skills [3, 4].

The childhood memory development happens during the first eight years in life. Childhood memory problems could lead to language delay, motor developmental problems, and poor academic performance. Therefore, it is necessary to investigate the factors that influence the memorial abilities of children [4–6].

Individuals differ in their cognitive skills mainly due to potential heritability and genetic influences [7] as well as from various environmental influences present during childhood. In addition to age and gender [2–5], factors such as socioeconomic status [6–8], parents’ education and occupation [9–12], nicotine exposure [13–15], area of residence [16], birth order among siblings [17], breakfast eating habits and culture [18], school setting [16–18] have been shown to be related to cognitive development in Western cultures. Most of these factors are likely reflecting the amount of cognitive stimulation and environmental conditions including social experiences and communications. These factors are also reflecting the child-rearing practices in the Western culture. In this research, we aimed to investigate the relationship of these variables to memory of children from the Middle East. Accordingly, we suppose that each culture has various parental styles that affect children’s behaviors, development and memorial abilities in different ways [19, 20]. The nature of children’s memory and other cognitive abilities and the relation between them is not well understood [17–20]. Furthermore, most of previous studies in this area investigated the effect of different variables on cognitive skills in general and not on memory skills in specific, where correlates of memory have not yet been examined in Middle-Eastern cultures. Therefore, further studies in this specific area are needed. We believe that children vary in their memory capabilities and that these variations affect their learning and academic performance [21, 22]. Studies have shown that memory abilities affect reading and mathematics skills, these abilities and skills could be reinforced in school, and could predict school readiness and academic success [23, 24]. Therefore, assessing children’s memory abilities could provide a picture of their strengths and weaknesses related to their school performance [25, 26]. The early identification of a group of children who are at high risk for poor academic performance would help therapists, teachers and parents in the early implementation of appropriate interventions, with the potential for positive long-term effects on school success and subsequent social and vocational opportunities [27, 28].

Nowadays, there are many standardized instruments used to assess different cognitive abilities. Examples of these instruments include Kaufman Assessment Battery for Children [29], Behavior Rating Inventory of Executive Functioning [30] and Lowenstein Occupational Therapy Cognitive Assessment [31]. However, it is very challenging to assess cogntive functioning outside Western cultures because these assemmnets were not standardized and validated to be used in Arabic speaking population. The Leiter International Performance Scale is an alternative to these instruments [32]. It was developed to be a comprehensive nonverbal diagnostic assessment to be sensitive to small differences in cognitive skills and allow early assessment of cognitive delays irrespective of language capacity. Thus, the Leiter-R appears to be a good choice for evaluating memory in populations from non-English speaking backgrounds including Jordan.

In summary, a group of 550 families were invited to participate in this project. The main objective of this research was to investigate whether sociodemographic variables, gender, age, and other variables related to parents would or not predict memory abilities in normally developing children.

## Method

### Participants

The Jordanian Ministry of Education provided authors with a list of private and public elementary schools (148 private schools and 140 public schools) in the area of North Jordan. This list involves schoolchildren from 5 to 9 years old. A multilevel stratified cluster sampling method was used to randomly select schools from the obtained list of elementary schools. The stratification was based on district, gender, and type of school. Some of the classes were co-educational in the selected public elementary schools whereas all classes were coeducational including both genders in the selected private schools. Then, investigators randomly (using simple random technique) selected classes from each participating school and included all students in classrooms. A total of 434 randomly selected children were included in the study. Any child with normal development, aged 5–9 years old, attended regular school was eligible to be part of the study. Children who diagnosed with neurological, developmental, or learning disability were excluded. Prior to data collection, 550 packages contained information about the purpose and procedures of the current study, written parental consent form, and self-reporting questionnaire were dispensed to the selected schools to be completed voluntarily by parents .

Of them, 70 (13%) parents refused to allow their children to be recruited whereas 46 (8%) children were not eligible.

The sociodemographic characteristics of enrolled students are presented in Table 1. Children’s aged 5 to 9 years old (mean = 8.16; SD = 1.9). Girls (57%) and students live in urban suburbs (54%) were more than boys and those live in rural areas respectively, with similar rates in public and private schools. Almost all of the children live with their parents (98%), of them, 51% of conveyed a family monthly salary lower than 6000 Jordanian Dinar yearly. About 59% of children reported having breakfast at home regularly, 64% slept at least 9 h every day, and 60% stated a GPA of 80 or above. According to Jordanian education system, The Grade Point Average (GPA) scores are from 35 to 100. A score of 90 to100 points is an excellent and a score of less than 50 points is a failure.

**Table 1:** Socio-demographic variables for study sample (*N* = 434)

Variable	N (%)
Age (Years)	
5–6.5	253(58)
6.6–9	181(41)
Mean ± SD	8.16 ± 1.9
Gender	
Male	189(43)
Female	245(57)
Area of living	
Urban	236(54)
Rural	198(46)
School type	
Public	214(49)
Private	220(51)
Students live with both parents	
Yes	427(98)
Student’s GPA	
50–69	69(16)
70–89	231(53)
90–100	133(31)
Family yearly income (Jordan Dinar)	
< 6000	223(51)
6000–12,000	120(28)
12,000–24,000	52(12)
> 24,000	39(9)
Child takes breakfast at home	
Yes	254(59)
Daily sleeping hours	
≥9 h	276(64)
< 9 h	158(36)

Moreover, Table 2 shows that about 6% of fathers were not employed and 65% of mothers were not working. About 33.3% of mothers had high school education whereas 41% of the fathers had bachelor’s degree. Additionally, more than half of fathers (62%) were cigarette smokers and only 5% of mothers were smokers. In the current study, a smoker was defined as having smoked more than 10 cigarettes inside home in one day for more than five years.

**Table 2:** Parents` variables for study sample (*N* = 434)

Variable	N(%)
Mother’s occupation	
Not employed	282(65)
Employed	
Military	19(4)
Hand-maker	26(6)
Administration	55(13)
Profession	52(12)
Mother’s level of education	
<high school	69(14.7)
High school	156(33.3)
2-years diploma	74(15.8)
Bachelor	106(22.6)
Graduate Degree	63(13.5)
Father’s occupation	
Not employed	28(6)
Employed	
Military	107(25)
Hand-maker	85(20)
Administrative	113(26)
Profession	101(23)
Father's level of education	
<high school	60(14)
High school	87(20)
2-years diploma	29(7)
Bachelor	178(41)
Graduate Degree	80(18)
Mother's smoking	
Yes	21(5)
Father’s smoking	
Yes	271(62)

### Demographic variables

A number of demographic variables related to participating children were measured as potential factors associated with memory including: age and order of the child, breakfast habit, place of residency, average hours of sleep daily, GPA in school, smoking status of parents, occupation of the parents, level of education of the parents, and total annual family income in Jordanian Dinar. Unlike other variables included in the analyses, GPA score could be affected by memory ability, not an influence on memory.

### Instrument

In the current study, authors used the Leiter International Performance Scale-Revised (Leiter-R) [32] to examine children’s cognitive abilities such as memory, attention, visualization, and reasoning. This instrument is reliable and valid with Chronbach’s alpha reliability coefficients ranged from 0.89 to 0.91% for visualization and reasoning battery and 0.76 to 0.88% for memory battery [32, 33]. The internal consistency (Chronbach’s alpha) reliability coefficients for the memory battery subtests of the Leiter-R in this study ranges from 71 to 87% (AP = 0.71%, IR = 0.83%, FM = 0.82%, RM = 0.85%, VC = 0.87%, SM = 0.82%, DP = 0.70%, DR = 0.74%). The scale was designed for children with any kind of communicative, hearing, or motor deficiencies and for bilingual individuals whose English is a second language. This instrument comprises 40 items divided equally into two subscales: (I) the subscale of memory and attention (MA); it comprises ten items testing non-verbal memory and attentional functioning (II) the subscale of reasoning and visualization (RV); it comprises ten items testing non-verbal cognitive skills related to spatial abilities, reasoning and visualization [32]. The visualization subsection contains tests for Paper Folding (PF), tests for Matching (M), tests for Figure Rotation (FR), tests for Form Completion (FC), Tests for Picture Context (PC) and tests for Figure Ground (FG). The reasoning subtests contains tests for Sequential Order (SO), tests for Repeated Patterns (RP), tests for Classifications (C), and tests for Design analogies (DA). The memory subtests contains tests for Delayed Recognition (DR), tests for Delayed Pairs (DP), tests for Association Pairs (AP), tests for Reverse Memory (RM), tests for Visual Coding (VC), tests for Immediate Recognition (IR), tests for Spatial Memory and tests for Forward Memory (FM). The attention subtests contain the tests of Attention Divide (AD) and tests of Attention Sustained (AS). For the purpose of the current work, the authors only analyzed the subjects’ composite scores in memory tests. As recommended by the inventor of the instrument, it is not necessary to give all subtests to all ages. Less number of subtests can be given for younger individuals and more subtests for older ones [32]. For instance, FM, RM, AS, SM, IR,VC, DR, DP, AP and AD items from the MA subtests and PF, SO, M, FC, FG, DA and RP items from the RV subtests were used for individuals with age ranges from 6 to 10 years.

Composite scores for each subtests of the Leiter-R’s instrument were computed using the standardized and normed composite scores as specified in the Leiter-R manual ([32], Appendix E). There are five composite scores for memory: memory screen (AP & FM) (normal score for all ages ranges from 44 to 155), associative memory (AP&DP) (normal score for ages 6–20 ranges from 50 to 151), memory span (FM, RM & SM) (normal score for ages 6–20 ranges from 42 to 157), working memory (FM, VC & SM) (normal score for ages 6–20 ranges from 38 to 161) and recognition memory (IR & DR) (normal score for ages 4 to 10 ranges from 48 to 150). A higher score means better cognitive (memory) performance.

### Translation procedure

Five bilingual college professors translated the manual of Leiter-R instrument into Arabic using backward-forward translation process [34], which is similar to that used for the Lowenstein Occupational Therapy Cognitive Assessment and Adolescent/Adult Sensory Profile. Inconsistencies in the translation of specific items were discussed until an agreement was reached. A pilot study was conducted in a group of twenty children for the purpose of increasing the readability if the initially translated version of the instrument. All items of the instrument were then revised accordingly and piloted to another group of twenty children. After that, investigators agreed that no further modifications were needed for the items of the instrument. The backward translation of the instrument from Arabic to English was performed by an individual who is fluent in Arabic and English language and not familiar with the original instrument. The backward translated instrument was assessed and approved by 10 bilingual college professors.

During the translation process, each translated item was given a score of 1 if it is similar to the original instrument and a score of 0 if it is not similar. The cut score for adequate translation was 0.8 which means that 80 % of the investigators accepted that the backward translation of the items reached the same meaning of the items in the original instrument. All items scored below 0.8 were modified and re-evaluated till they reach the cut score or better.

### Procedure

In order to perform this descriptive, cross-sectional design study, authors obtained ethical approval from Jordanian Ministry of Education, Institutional Review Board and Deanship of Research at Jordan University of Science and Technology. Before the process of data collection, signed, voluntary written parental consent was obtained. Memory abilities and skills in participating children were measured by using the Leiter International Performance Scale-Revised (Leiter-R). The principle investigator intensively trained four research assistants on the process of data collection as well as the process of administering and scoring the instrument. Twenty school children (5–9 years) were assessed and scored by the each research assistants until 98% of inter-rater reliability was achieved. The assessment of recruited children was carried out in a quiet room in their schools and took sixty to ninety minutes in an average.

### Data processing and statistical analyses

The Data was saved using the program of SPSS Version 20 (SPSS, 2012). Then, data was cleaned, coded, and analyzed using the same program. Descriptive statistics including means (M) and standard deviations (SD) were performed to describe socio demographic variables and subjects’ memory abilities using the Leiter-R instrument.

The relationship between measured demographic variables and other possible factors related to memory in children and memory abilities, as measured by Leiter-R, in the study sample was performed using the analysis of multivariate linear regression model. The backward elimination method was applied to all variables entered to the model. All related variables were entered into the model using the backward elimination method. The level of significance was set at the probability level of *P*-value equal or less than 0.05.

## Results

Table 3 represents the composite scores of the Leiter-R Memory battery, including memory screen, associative memory, memory span, memory process and recognition memory, as a function of age. The youngest children had the lowest composite scores (M = 38.54; SD = 2.65, in memory process); the highest average score (M = 104.95, SD = 28.55, in associative memory) were produced by the oldest children. These scores represent normal memory, as indicated in the Leiter-R manual.

**Table 3:** Leiter-R composite scores average (SD) in Memory battery by subjects’ age groups

Age	Memory Screen Mean (SD)	Associate Memory Mean (SD)	Memory Span Mean(SD)	Memory Process Mean(SD)	Memory Recognition Mean(SD)
5–5.9 years	50.43(5.78)	51.41(8.55)	42.38(2.30)	38.54(2.65)	67.70(20.69)
6–6.9 years	61.11(17.92)	68.33(28.76)	45.28(5.02)	41.81(5.65)	80.50(16.76)
7–7.9 years	81.75(15.74)	103.22(25.87)	51.00(3.15)	47.44(1.32)	95.33(10.58)
8–9 years	89.80(18.99)	104.95(28.55)	57.60(5.34)	55.55(8.19)	102.58(5.45)

Table 4 represents the results of the linear regression analyses. The following variables accounted for 48.9% of the variance (df = 29) (St error = 9.137) in the mean average of all memory composite scores, including memory span, memory screen, memory recognition, associative memory and memory process: smoking status of father, family total income per annum, having regular breakfast at home, mother’s occupation and sleeping of nine hours and more daily. The average memory composite score improved by 5.44 points for each year increase in the child’s age, 2.53 points with increase in mother’s occupation level, 3.08 points when the child had breakfast every morning and 4.51 points when the child sleeps daily nine hours and more. Conversely, the average composite score of memory functions declined by 2.02 points for each increase in the annual family income and 3.93 points if the child’s father is a smoker.

**Table 4:** Predictors of memory abilities (*n* = 434)

Memory Battery		R^2^ = 0.489		
Significant Independent Variables^a^	B	T-value	Beta	P
Eating breakfast regularly	13.649	3.083	0.191	.031
Mother’s occupation	10.64	2.534	0.322	.042
Family yearly income(Jordan Dinar)	−11.05	−2.018	−0.249	.023
Father’s smoking status	−18.65	−3.933	−0.763	.009
Daily sleeping hours	19.72	4.506	0.896	.003

^a^Included variables (all variables): family annual income, child’s gender, child’s type of school, child’s birth order among siblings, child lives with both parents, mother’s smoking, father’s smoking, family area of residence, father’s education, mother’s occupation, mother’s education, father’s occupation, child’s GPA, child’s daily sleeping hours and child’s eating breakfast at home before school

## Discussion

The current study indicates that different factors are largely associated with memory abilities including mothers’ level of occupation, family yearly income, smoking status of father, having regular breakfast and going to public school. In contrast, father’s occupation, mother’s smoking status, parent’s education, area of residency, school type, number of sleeping hours, birth order and GPA are not related to memory abilities of the study sample.

### Mothers’ occupation

The study found that memory abilities improved with increased level of mother’s occupation. For example, a child of a physician mother would be expected to have better memory abilities than a child of a hand maker. This finding is supported by previous research [10, 12, 35, 36] and attributable to the fact that, in Jordan culture, increased level of mother’s job is related to a higher level of mother’s education and that the mother is most probably married to an educated father with a high level of occupation. For example, a previous research suggested that low level of parents’ education would be strongly related to worse performance of children (10–13 years) in working memory [10]. In addition, it was found that a high level of mothers’ education was strong predicator of the memory’s abilities and skills in young children. This result was reported by a large cohort and longitudinal study [12]. Not only that, it was found that the occupation of mothers influence the communication style between the mother and the child and that affects the different memory skills in children. The majority of the mothers and 6 % of the fathers were not working in this current study. Therefore, it is very important to promote and encourage policies and programs that boost employment for parents in Jordan. Also, high quality day cares and parenting programs are highly recommended for early stimulation and interventions, and hold promises for improving memory skills among children with special needs [9, 10].

### Smoking of father

The present study found that memory scores were better for children with non-smoking fathers than children with smoking fathers. Mothers’ smoking was not affecting children’s memory because most mothers were not smokers as this practice is not socially acceptable among women in Jordanian culture. Available literature found that smoking status of parents was negatively associated with memory and academic performance of children [14, 15, 37, 38]. For example, a large study found that children with different memory disabilities were more exposed to second hand smoking than their peers [39] highlighting the negative effect of nicotine exposure on working memory [14]. Moreover, tobacco consumption is an enormous health burden among Jordanians, who they report one of the highest rates of smoking among males in the area of the Middle East [40]. A previous study found that the prevalence of smoking among Jordanian boys in grades 7 and 8 was 35.6% [41]. About 50% of the sample were shisha smokers [41]. A more recent national study on Jordanian adolescent boys and girls found that 30 % smoked both waterpipe and cigarettes and around 58 % smoked only cigarettes [42]. Sixty five percent of the children’s fathers in the current study reported smoking. Therefore, there is a huge need for increasing the awareness of the negative effects of second hand smoking on children through the initiation of smoking cessation programs among the population of Jordan.

### Eating breakfast regularly

Similar to previous studies, in this study we have found that children with regular breakfast habit had better memory abilities. The consumption of nutritious breakfast is very crucial for children health and development [43]. The improvement of short term hunger positively impacted memory, information processing, and participation in class rooms [43, 44]. Available literature concluded that malnutrition has harmful effect on working memory, children behaviors, deprived brain essential nutrients, academic performance, and even undermining later adult productivity [17, 45–50]. Similarly, glycemic index and glycemic load of breakfast can forecast memory functioning and mood in young children [49, 50] as well as maintain attention and memory during the morning [47]. In fact, Basch [51] concluded that contribution in breakfast program in schools among urban minority children had favorable influence on memory, academic achievement, and school absences [51]. Hence, the results of the this and previous studies indicate the essential of having breakfast regularly for children and the bad effect of poor nutrition. Also, additional research is needed to investigate the effect of having different foods for breakfast on child’s memory. In addition, Jordanian private and governmental schools do not provide breakfast for their students and around 40 % of children in this study do not eat breakfast at home. Therefore, it is very necessary to recommend school breakfast programs for the ministry of education in Jordan.

### Family yearly income

The study found that memory abilities diminish with the increase in family yearly income. As such, a child with low family yearly income has a better score in memory than a child with higher family yearly income. This result was controversial and not clearly supported by previous research. Previous literature reported that memory functions of school children were positively associated with high family yearly income and socioeconomic characteristics [6, 19, 35, 36, 52], whereas, other research found working memory has no clear and direct effect on socioeconomic status [7, 8]. The finding that family yearly income was indirectly (negatively) related to memory abilities in the current study may be attributable to many reasons. First, previous studies focused on investigating the effect of sociodemographic on general cognitive abilities without differentiating between visualization, reasoning, attention or memory [6, 35, 36]. Researches were also focusing mainly on older children and not very young children and that could have affected the results [6]. Second, almost more than 50 % of participating children had a low annual income for the families. Therefore, a noticeable difference was found in the proportion of children in the high socioeconomic status category compared to low socioeconomic status category. Third, the current study investigated the effect of annual family income in specific, whereas previous research investigated the effect of socioeconomic status in general. In the country of Jordan, there are some children who might live with not-employed but highly educated parents, live in very good housing conditions, live in prestigious neighborhoods and go to fancy schools and though having low annual family income, because these families may depend on their grandparents on other financial supports. Therefore, there is a need for future research to further investigate the effect of income and culture on different memory abilities and skills among different age groups and cultures.

### Hours of daily sleeping

In the present study a significant relationship between child’s sleeping hours and memory abilities was reported. Previous researches have shown that sleep deprivation and low number of sleeping hours negatively affects memory [16, 18, 53, 54]. Critical review by Kopasz et al. [53] found that sleep aids children’s working memory, memory consolidation, and performance in complex task decline strongly after sleep deprivation. Another experimental study on 33 typically developing children (6–12 years) found that sleep quality and duration is necessary for memory consolidation and plays important role in children’s academic performance [54]. Further research examining the influence of sleep on memory and attention of school children in larger samples, with sleep limited hours, is needed.

### Other factors

The current study found that some factors were not associated with memory, however, available literature found associations with these factors, which include child’s GPA [21, 22, 24], area of residency [16], numbers of sleeping hours [53, 54], and birth order of child in the family [17, 18]. For example, previous research found that memory could be directly related to schooling success and the child academic performance at school [21–24]. The finding that child’s GPA was not significantly related to memory in this study could be attributed to the fact that GPA score could be affected by memory ability, not an influence on memory.

Although memory and GPA all reflect child’s overall capabilities, they usually vary in their concepts; they cannot predict each other reliably, and they cannot be considered interchangeably [22, 55, 56]. GPA assesses the school children’s academic abilities, which may be influenced by extraneous factors. Another possible explanation for the non-significant results of GPA is that the researchers excluded children with learning, developmental, or neurological disability, thus recruited children are likely to have with-in normal range of memory abilities, thus changes in GPA scores were not significant to memory.

Nonetheless, these factors may not affect memory tests. These findings suggest that factors affecting academic performance and memory should be more investigated in future research and followed by discussing suitable intervention strategies.

Additionally, no significant differences found in memory of the children neither in public and private schools nor in rural versus urban areas, however, some literature found inconsistent results [16, 57]. Our results can be attributed to the fact that the majority of schools in the private sectors teach school curricula similar to the public ones. The ministry of education in the country of Jordan more budgets and gives special care for public elementary schools, making these schools as good as private elementary schools. In addition, children who attend private schools belong commonly to moderate to high socioeconomic status and usually their mothers have a job thus spend less time teaching their children. On the other hand, most of children who are enrolled in public schools belong to families with poor socioeconomic status and usually have mothers as housekeepers and thus have an active role in their children’s memory functioning. Moreover, our results may be attributed to the fact that urban and rural areas in Jordan are exposed to similar environmental stressors (noise, pollution, etc.) and are provided with similar playground facilities and similar child educational system as provided by the ministry of education in Jordan.

Finally, additional studies examining the influence of other variables on memory abilities are needed. These include the presence of other medical condition, the effect of air and water pollution, the effect of breast feeding [58, 59], parenting style [60], living in safe environment [61, 62], birth weight [63, 64], and the amount of child’s physical activity.

The school and occupational success later in life are highly depending on early detection of different memory problems [4, 28]. Future research should study the effect of different intervention and rehabilitation programs. These programs would include the effect of physical activity, special diets and breast feeding on memory skills [65, 66].

### Limitations

Although there are several important implications for the current study, some limitations should be addressed. First, the reported results are derived from a cross-sectional datan, as it is difficult to make causal relation. Moreover, the cluster sampling design could have led to inadequate effect and sample size. However, the random sampling of the clusters, including classes, schools as well as students might have overcome the issues with the cluster design. Furthermore, the participating schools were recruited from the northern part of Jordan, and this might limit the possibility for generalizing the results to other part of the country. Another issue was regarding the analysis of regression of the five predicting variables. The analysis showed about 36 % of variance in memory is explained, whereas other factors not measured in this study, could have explained the variance in the memory variables. Finally, there is a possibility of producing subjective data through the use of self reported questionnaires. Therefore future research could use objective data and different statistical methods to confirm the findings of this study.

## Conclusions

This study found that age, mother’ occupation, smoking status of father, family income, having regular breakfast and daily sleeping hours correlated with memory function of children (5–9 years). Memory abilities have been found to be correlated with different variables such as environment, culture, sociodemographic, and others, thus affecting the amount of training, opportunities and care provided to children. The understanding of these interactions will enable professionals to assess and screen different type of memory skills, establish treatment objectives and methods, and accommodate abilities and limitations of children.

## Abbreviations

AD: Attention Divided
AM: Attention and Memory
AP: Association Pairs
AS: Attention Sustained
C: Classification tests
DA: Design analogies test
DP: Delayed Pairs
DR: Delayed Recognition
FC: Form Completion tests
FG: Figure Ground test
FM: Forward Memory
FR: Figure Rotation
GPA: Grade Point Average
IR: Immediate Recognition
Leiter-R: Leiter International Performance scale-Revised
M: Matching tests
PC: Picture Context tests
PF: Paper Folding Tests
RM: Reverse Memory
RP: Patterns
SM: Spatial Memory
SO: Sequential Order tests
VC: Visual Coding
VR: Visualization and Reasoning
